# High Affinity Nanobodies against the *Trypanosome brucei* VSG Are Potent Trypanolytic Agents that Block Endocytosis

**DOI:** 10.1371/journal.ppat.1002072

**Published:** 2011-06-16

**Authors:** Benoît Stijlemans, Guy Caljon, Senthil Kumar A. Natesan, Dirk Saerens, Katja Conrath, David Pérez-Morga, Jeremy N. Skepper, Alexandros Nikolaou, Lea Brys, Etienne Pays, Stefan Magez, Mark C. Field, Patrick De Baetselier, Serge Muyldermans

**Affiliations:** 1 Laboratory of Cellular and Molecular Immunology, Vrije Universiteit Brussels, Brussels, Belgium; 2 Department of Molecular and Cellular Interactions, VIB, Brussels, Belgium; 3 Department of Animal Health, Unit of Veterinary Protozoology, Institute of Tropical Medicine Antwerp (ITM), Antwerp, Belgium; 4 Department of Pathology, University of Cambridge, Cambridge, United Kingdom; 5 Laboratory of Molecular Parasitology, IBMM, Université Libre de Bruxelles, Gosselies, Belgium; 6 Multiimaging Centre, Department of Physiology Development and Neuroscience, University of Cambridge, Cambridge, United Kingdom; 7 Department of Molecular and Biochemical Pharmacology, Vrije Universiteit Brussels, Brussels, Belgium; Seattle Biomedical Research Institute, United States of America

## Abstract

The African trypanosome *Trypanosoma brucei*, which persists within the bloodstream of the mammalian host, has evolved potent mechanisms for immune evasion. Specifically, antigenic variation of the variant-specific surface glycoprotein (VSG) and a highly active endocytosis and recycling of the surface coat efficiently delay killing mediated by anti-VSG antibodies. Consequently, conventional VSG-specific intact immunoglobulins are non-trypanocidal in the absence of complement. In sharp contrast, monovalent antigen-binding fragments, including 15 kDa nanobodies (Nb) derived from camelid heavy-chain antibodies (HCAbs) recognizing variant-specific VSG epitopes, efficiently lyse trypanosomes both *in vitro* and *in vivo*. This Nb-mediated lysis is preceded by very rapid immobilisation of the parasites, massive enlargement of the flagellar pocket and major blockade of endocytosis. This is accompanied by severe metabolic perturbations reflected by reduced intracellular ATP-levels and loss of mitochondrial membrane potential, culminating in cell death. Modification of anti-VSG Nbs through site-directed mutagenesis and by reconstitution into HCAbs, combined with unveiling of trypanolytic activity from intact immunoglobulins by papain proteolysis, demonstrates that the trypanolytic activity of Nbs and Fabs requires low molecular weight, monovalency and high affinity. We propose that the generation of low molecular weight VSG-specific trypanolytic nanobodies that impede endocytosis offers a new opportunity for developing novel trypanosomiasis therapeutics. In addition, these data suggest that the antigen-binding domain of an anti-microbial antibody harbours biological functionality that is latent in the intact immunoglobulin and is revealed only upon release of the antigen-binding fragment.

## Author Summary

Haemoparasites, such as African trypanosomes, have developed potent immune evasion mechanisms to avoid antibody-mediated elimination. Consequently, trypanosome surface antigen-specific immunoglobulins in the absence of complement are non-trypanocidal. In contrast, certain monovalent nanobodies (Nb), monomeric antigen-binding domains derived from camelid Heavy-Chain Antibodies (HCAb) and which have a much lower molecular weight (15 kDa) than classical antibodies (150 kDa), efficiently lyse trypanosomes both *in vitro* and *in vivo*. This is surprising as classically immunoglobulin effector functions are mediated via the Fc-domain, which is absent from the Nb. We demonstrate that the Nb-mediated trypanolysis depends on the low molecular weight, monovalency and high affinity and is associated with loss of motility, a major block to endocytosis, energy depletion and cell death. Overall, targeting the parasite surface with low molecular weight, high affinity Nbs is sufficient to exert a direct therapeutic action. Therefore, the exploitation of Nbs against African trypanosomiasis represents a novel therapeutic strategy. Furthermore, demonstration that a high affinity antigen-binding Nb or Fab fragment lacking an effector domain (i.e., Fc-domain or an attached toxin) can exert a direct biological function, suggests that intact antibodies likely harbour latent functionality which only become revealed upon removal of the Fc-domain.

## Introduction

Trypanosomatid protozoan parasites cause many important diseases, including African sleeping sickness in humans and Nagana in domestic livestock in sub-Saharan Africa [1], [2]. These organisms, like many other successful pathogens, have evolved sophisticated mechanisms for immune evasion [3]. A prominent strategy among African trypanosomes, facilitating chronic persistence in the host bloodstream and lymphatic system, relies on antigenic variation [4].

The major trypanosome surface antigen is the immunogenic variant-specific surface glycoprotein (VSG) present at ∼10^7^ copies per cell and representing ∼90% of the total cell surface proteins [5]. This dense VSG coat is envisaged as functioning as a physical barrier, impeding antibody recognition of invariant surface epitopes. By repeatedly switching the VSG coat, antibodies that would recognise trypanosomes, leading to their elimination, are evaded [4], [6]. Further, trypanosomes can reverse antibody-mediated agglutination in a protein synthesis-dependent manner [7], and also defend themselves by efficient internalisation of antibody-VSG complexes [8], [9], [10], [11], delaying elimination by antibody-dependent complement lysis [12], [13]. Furthermore, several groups have reported that antibody-induced VSG shedding may contribute to protection against antibody-mediated removal [7], [14], [15].

*Trypanosoma brucei*, in common with other trypanosomatids, restricts membrane exchange between the surface and endomembrane compartments to an invagination of the plasma membrane, the flagellar pocket (FP), which is contiguous with the pellicular and flagellar membranes [13], [16]. The FP comprises ∼5% of the total cellular surface and lacks the subpellicular microtubules [17], [18]. The lumen of the FP contains an electron dense carbohydrate rich matrix and is bounded by a hemidesmosome-like zone around the neck of the pocket. Solution macromolecules such as antibodies have to transit the hemidesmosomal zone and the matrix to enter the FP. The VSG density is similar at the luminal face of the FP membrane and the bulk plasma membrane, but many other proteins such as macromolecular receptors are enriched within the FP membrane and virtually absent from the cell surface (Field et al. [13]).

In the bloodstream stage of the trypanosome, the cell surface turnover is exceptionally high [12]; exceeding rates reported for macrophages and fibroblasts [19] and is sufficient to cycle the entire surface in under 15 minutes [20], [21]. While the biological significance of this high and developmental-stage specific activity is unclear, it likely contributes to a mechanism for recovering VSG and/or eliminating anti-VSG immunoglobulins bound to the surface of living parasites [12].

The difference in recycling efficiency of VSG and other surface proteins is due in part to differential trafficking through the endocytic pathway. For instance, transferrin is liberated from the parasite transferrin receptor in an acidic compartment, possibly the late endosome or the lysosome, whereas VSG is recycled via early and recycling endosomes [8], [20]. Following clathrin-dependent endocytosis at the FP, VSG is separated from bound antibodies in sorting endosomes and recycled to the parasite surface while the antibody is directed to a distinct pathway for degradation [8], [20], [22], [23], [24]. These distinct endosomal populations have been classified depending on the presence of several key components of the vesicle transport system i.e. Rab GTPases and other markers [25]. Specifically, early/sorting endosomes play a role in fluid-phase and transporter-mediated endocytosis and contain Rab5A or 5B [26], whereas the recycling endosomes mainly contain Rab11 [24].

The dense packing of VSG at the parasites' surface prohibits recognition of conserved membrane proximal VSG epitopes by antibodies. To potentially circumvent this we introduced camelid IgG-derived 15 kDa nanobodies (Nb), representing the intact antigen-binding domains of the unique camelid IgG2 or IgG3 90 kDa heavy-chain antibodies that are devoid of light chains (HCAb) [27]. The monomeric Nbs have dimensions of ∼4×2.2 nm and offer several advantages over antigen-binding fragments derived from classical antibodies [28]. High-affinity antigen-specific Nbs can be readily obtained from an immunised camelid and selected by phage display. They are also very robust and can be engineered efficiently into larger constructs to confer novel functionality and broaden their utility [29]. Moreover, several VSG-specific Nbs, directed towards distinct regions of the VSG molecule have already been identified, of which one targets a conserved VSG epitope that is on live trypanosomes inaccessible for larger antibodies [30], [31].

We now report that Nbs recognizing VSG isotype-specific epitopes are competent in lysing parasites both *in vitro* and *in vivo*. These trypanolytic Nbs rapidly arrest cell motility, block endocytosis, cause FP swelling, collapse mitochondrial membrane potential and exhaust ATP, ultimately leading to parasite death. However, the Nbs become non-lytic when reconstituted into HCAbs in the absence of complement. Further, polyclonal antibodies directed against VSG recognise live trypanosomes without any detectable toxicity in the absence of complement, whereas proteolytically derived monovalent Fab or Nb antigen-binding fragments are trypanolytic. These data suggest that the antigen-binding fragment of an antibody harbours biological functions which remain latent in the intact immunoglobulin.

## Results

### Identification of nanobodies that lyse *Trypanosoma brucei* AnTat1.1 parasites

**Figure 1:**
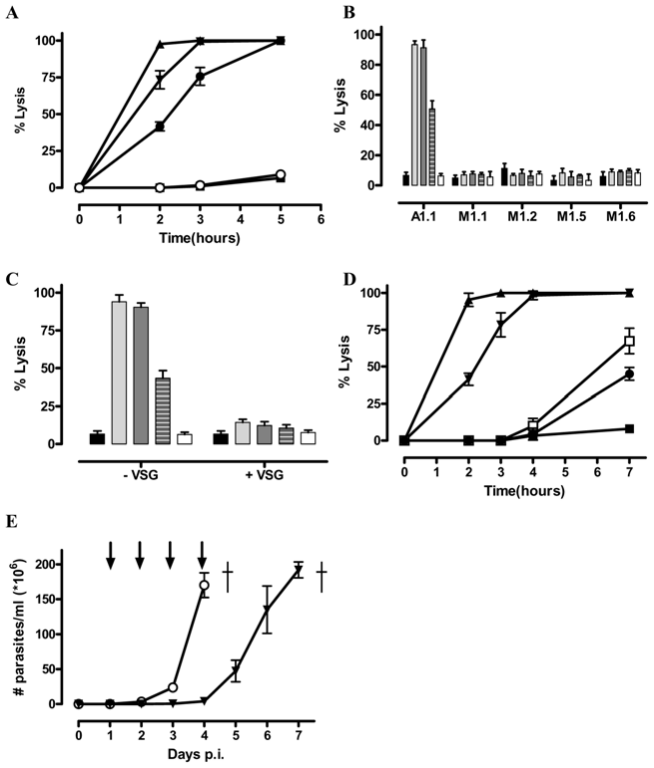
Identification of trypanolytic Nbs. 2×10^5^ monomorphic T. brucei parasites were kept in HMI-9 buffer at 37°C and counted hourly. A) Percent of lysed parasites at different times after adding 1 µg Nb_An05 (▴), Nb_An06 (•), Nb_An46 (▾), Nb_An33 (○) (VSG∶Nb ratio is 1/20) and a control with no Nb (▪). B) Percent lysis of antigenically distinct trypanosomes (AnTat1.1, MiTat1.1, MiTat1.2, MiTat1.5 and MiTat1.6) after two hours incubation with 1 µg Nb_An05 (light grey bars), Nb_An06 (dashed grey bars), Nb_An46 (dark grey bars), Nb_An33 (white bars) (VSG∶Nb molar ratio of 1∶20) and a control with no Nb (black bars). C) Inhibition of Nb-mediated trypanolysis using three-fold molar excess of AnTat1.1 VSG after two hours incubation with 1 µg Nb_An05 (light grey bars), Nb_An06 (dashed grey bars), Nb_An46 (dark grey bars), Nb_An33 (white bars) (VSG/Nb molar ratio of 1/20) and a control with no Nb (black bars). D) Percent lysed parasites after incubation with Nb_An05 at 1 µg (▴), 0.5 µg (▾), 0.1 µg (□), 0.05 µg (•) and control with no Nb (▪). These data are typical results from three independent experiments performed in triplicate (±SD). E) *In vivo* effect of Nbs on parasitemia development and survival of C57Bl/6 mice infected with virulent monomorphic AnTat1.1A parasites, injected i.v. with Nb_An46 (▾), Nb_An33 or without Nb treatment (○). Antibody injections were given at daily interval, starting from day one until day four post-infection (arrows). (†: indicates all mice died).

Previously several monoclonal Nbs against *Trypanosoma brucei* AnTat1.1 were isolated by panning of a phage-displayed nanobody (Nb) library from lymphocytes of a VSG-immunised camelid [30]. Some of these Nbs appear to be highly specific for the AnTat1.1 VSG, while others exhibit cross-reactivity towards a wide variety of distinct VSGs. Surprisingly, the AnTat1.1 VSG-specific Nbs (i.e. Nb_An05, Nb_An06 and Nb_An46) provoke efficient lysis of AnTat1.1 parasites within five hours (Fig. 1A). The specificity of this Nb-mediated trypanolysis is further confirmed, firstly by demonstrating that *Trypanosoma brucei* strains expressing MiTat1.1, MiTat1.2, MiTat1.5 and MiTat1.6 VSGs are not lysed (Fig. 1B) and secondly, via inhibiting trypanolytic activity by pre-incubation with a three-fold molar excess of purified soluble AnTat1.1 VSG prior to parasite challenge (Fig. 1C). Furthermore, using the most potent trypanolytic Nb, i.e. Nb_An05, it is noted that this lysis is dose-dependent (Fig. 1D). Similar observations were obtained for Nb_An06 and Nb_An46, but with different kinetics. Under identical assay conditions, Nb_An33, which cross-reacts with multiple *T. brucei* VSGs, had no significant effect on parasite viability.

To evaluate the therapeutic potential for Nbs *in vivo*, mice were infected with virulent monomorphic AnTat1.1A parasites and treated with lytic or non-lytic Nbs at daily intervals, starting at day one and progressing to day four post-infection. Untreated mice and those treated with the non-lytic Nb_An33 reached extremely high levels of parasitaemia within four days (Fig. 1E). In contrast, mice treated with trypanolytic Nb_An05, 06 or 46 had no detectable parasites during the entire treatment period. However, upon interruption of the Nb treatment, parasites reappeared in the blood and proliferated to ∼2×10^8^ parasites/ml by day seven post infection (Fig. 1E).

### Trypanolytic nanobodies alter trypanosome morphology

**Figure 2:**
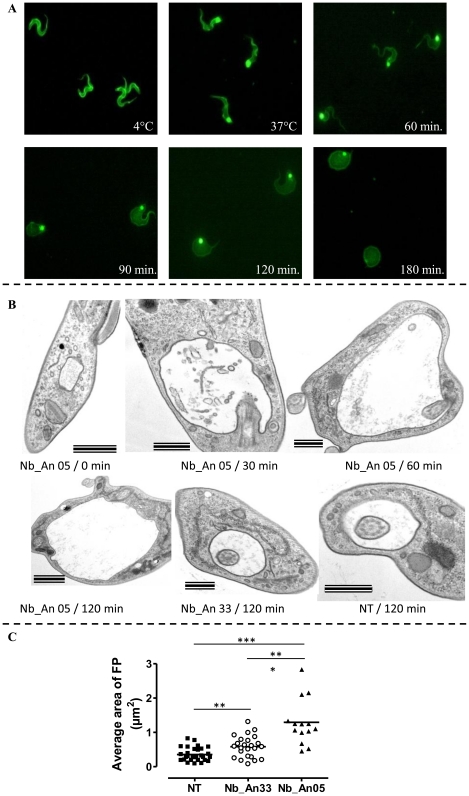
Immunofluorescence picture illustrating the effects of ALEXA-labelled Nb_An05 on AnTat1.1 parasites. A) Parasites were incubated at 4°C (upper right panel) and 37°C (upper middle panel). The morphology of trypanosomes kept at 37°C in the presence of Nb_An05 after 60, 90, 120 and 180 minutes incubation are shown. For each time point 10^6^ parasites were incubated with 1 µg Nb (VSG∶Nb ratio is 1∶4). B) Electron microscopy on sections of AnTat1.1 parasites incubated with Nbs for different time periods. Trypanosomes (10^7^) were treated with 10 µg of Nb_An05 or Nb_An33 (ratio VSG∶Nb equals 1∶4) or left untreated (NT: not treated) for the indicated periods, fixed and processed for EM. Images of ultrathin sections are shown. Nb_An05 causes enlargement of the FP. Scale bars are 500 nm, except in Nb_An05/0 min and Nb_An05/120 min panels where bars are 1 µm. Notice the accumulation of vesicles in the FP lumen and the enlargement of this compartment in Nb_An05 treated trypanosomes compared to Nb_An33 treated or untreated parasites. C) The average size of the FP transversal lumen surface, including the flagellum, after 60 minutes incubation in the presence (Nb_An05 (▴), Nb_An33 (○)) or absence of Nbs (▪). (**: p-value<0.005 and ***: p-value<0.0001).

The progress of the Nb-mediated trypanolysis was followed by immuno-fluorescence. Addition of ALEXA-labelled Nb_An05 to AnTat1.1 trypanosomes, maintained at 4°C, stained the parasites over their entire surface (Fig. 2A upper left panel), whereas at 37°C, the stain concentrated rapidly in the flagellar pocket (FP) (Fig. 2A, upper middle panel). Monitoring trypanosomes at 37°C revealed that the parasites were rapidly hampered in their mobility, within minutes following the addition of lytic Nbs, and finally became immobile. Subsequently, morphological abnormalities became noticeable whereby a gradual swelling takes place until a globular shape is adopted was attained. These parasites exhibited progressively weaker Nb_An05 surface staining (Fig. 2A), and eventually lysed. Although the kinetics are slightly distinct, highly similar observations were obtained for Nb_An06 and Nb_An46.

Transmission electron microscopy on ultrathin sections was used to investigate these morphological changes further (Fig. 2B). The early and prominent feature was emergence of a large vacuole, determined to be the FP on account of morphology, presence of a flagellum, and kinetoplast, matrix material in the lumen and position within the cell [13], [32]. No FP enlargement was observed in the absence of Nbs (Fig. 2B). The FP surface area after one hour incubation was significantly increased in the presence of trypanolytic Nb_An05 as compared to control cells either treated with non-lytic Nb_An33 or no Nb (Fig. 2C). Interestingly, a small but significant increase in FP surface area was observed between parasites incubated with non-lytic Nb_An33 compared to untreated parasites.

### Trypanolytic nanobodies, endocytosis and disrupted energetics

**Figure 3:**
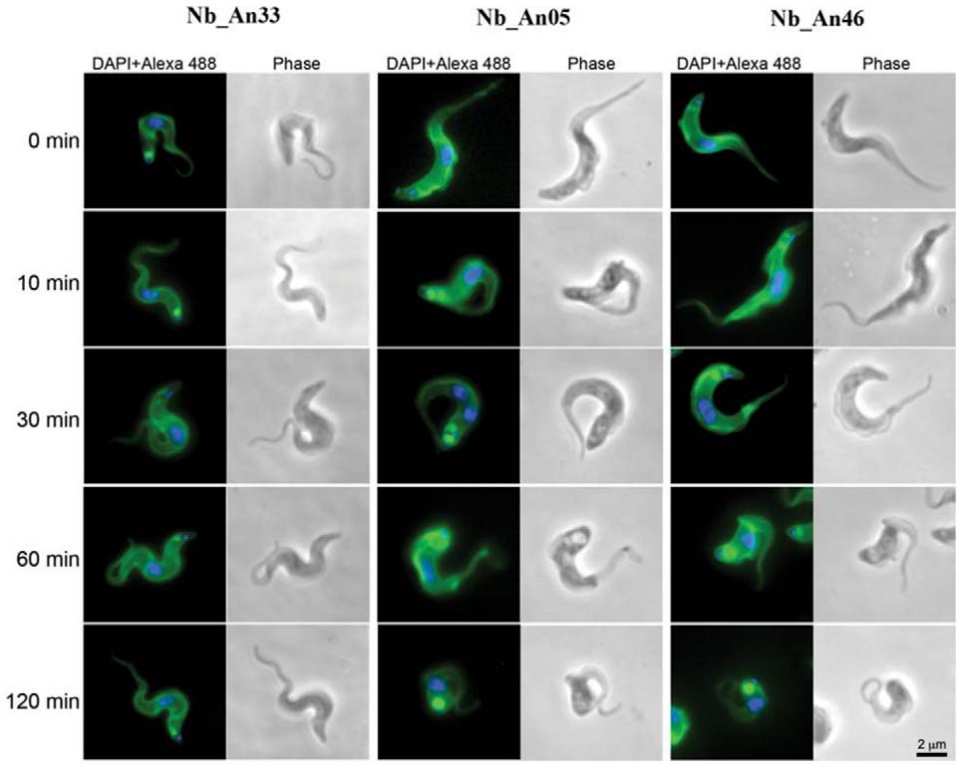
Trypanolytic Nbs accumulate in the FP. Uptake of ALEXA-488 labelled Nbs (Nb_An33, Nb_An05 and Nb_An46) by trypanosomes over a two hour period at 37°C. All ALEXA-488 labelled Nbs stain the entire surface of the parasite, but only ALEXA-488 labelled trypanolytic Nb_An05 and Nb_An46 stain the FP intensively compared to control Nb_An33. The FP is enlarged in the presence of trypanolytic Nb_An05 and Nb_An46 compared to control Nb_An33. ALEXA-488 labelled Nbs are shown in green, nuclei and kinetoplast are stained with DAPI and in blue. For each time point 10^6^ parasites were incubated with 1 µg Nb (VSG∶Nb ratio is 1∶4). Swollen flagella can be seen emerging from the neck of the structure containing Nb in panels 2 and 3 confirming assignment as the FP organelle. FP, kinetoplast DNA and mitochondrial lumen are indicated by fp, k and m respectively. Scale bar is 500 nm.

Nb_An05 and Nb_An46 stain the FP more intensively when compared to the non-lytic Nb_An33 (Fig. 3). Furthermore, in the presence of lytic Nbs, especially Nb_An05, the FP is greatly enlarged as compared to the control Nb_An33 where the FP size remains essentially unaltered. Both, enlargement of the FP and failure to traffic the Nbs into internal compartments suggest that endocytosis is blocked at the FP.

**Figure 4:**
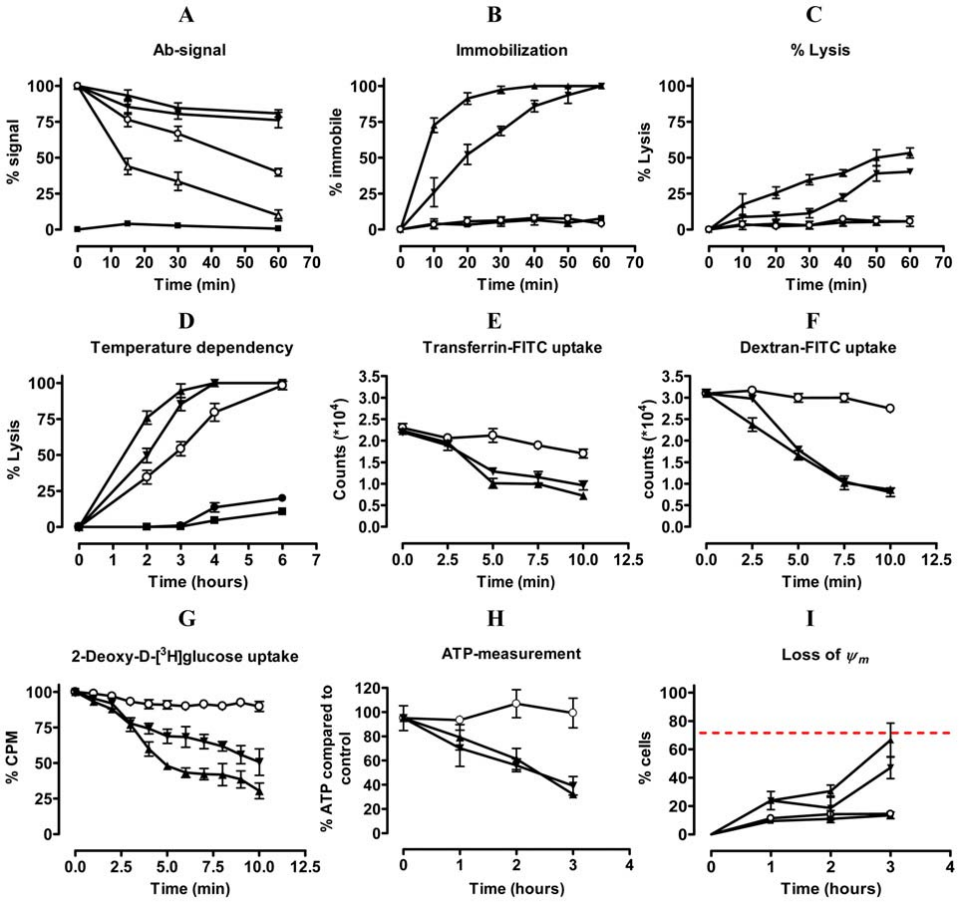
Unravelling the mechanism of trypanolysis. A) Pulse-chase analysis of clearance of ALEXA-labelled Nbs (Nb_An33 (○), Nb_An05 (▴), Nb_An46 (▾), irrelevant Nb (▪)) and polyclonal rabbit anti-VSG IgG (▵)) from the parasite surface was monitored for one hour. Percent antibody (Ab) signal represents the percent of signal (mean fluorescence) compared to time zero (starting point; 100%). B and C) Parasite immobilisation and percent lysis, respectively, during the first hour following addition of Nbs (Nb symbols as in A). D). Percent parasites lysed over a six hour period at 4°C (•), 15°C (∘), 23°C (▾) or 37°C (▴) in presence of Nb_An05 or with no Nb (▪). E) FITC-labelled transferrin uptake by AnTat1.1 parasites during the initial ten minutes following addition of different Nbs (Nb_An33 (○), Nb_An05 (▴) and Nb_An46 (▾)). F) FITC-labelled Dextran uptake by AnTat1.1 parasites during the initial ten minutes after addition of different Nbs (Nb symbols as in (E)). G) Percentage of 2-deoxy-D-[3H]glucose-uptake by AnTat1.1 parasites during the initial 10 minutes of addition of Nbs (Nb symbols as in (E)). H) ATP levels in the presence of Nbs over a three hour period (Nb symbols as in (E)). I) Mitochondrial membrane potential (Δψ_m_) determined using the MiToPT™ JC-1 kit. Parasites in the presence of Nbs ((Nb_An33 (○), Nb_An05 (▴) and Nb_An46 (▾)) were analysed after different times exposure. The red line represents the Δψ_m_ of parasites in the presence of a membrane depolarizing agent (CCCP), i.e. positive control (•), for 60 minutes. The negative control (□) consists of parasites in the absence of Nb. The data are typical results from three independent experiments performed in triplicate (±SD). For each experiment 2×10^5^ parasites were incubated with 1 µg Nb (VSG∶Nb molar ratio of 1∶20) or 10 µg rabbit anti-VSG IgG, except for 2-deoxy-D-[3H]glucose-uptake experiments where 2×10^6^ parasites were incubated with 2 µg Nbs (VSG∶Nb molar ratio of 1∶4) and ATP measurement where 10^7^ parasites were incubated with 10 µg Nb (VSG∶Nb molar ratio of 1∶4).

A flow cytometry-based pulse-chase experiment indicated greatly impaired clearance of Nb-VSG complexes when parasites are incubated with trypanolytic Nbs compared to non-lytic or conventional anti-VSG antibodies (Fig. 4A). Interestingly, clearance of the non-lytic Nb_An33 was slower than conventional anti-VSG IgGs. Since motility plays a key role in clearance of antibody-bound VSG, we evaluated the effect of trypanolytic Nbs on parasite motility. Within 10–60 minutes of exposure to lytic Nbs greatly reduced parasite motility occurs, which precedes cell lysis (Fig. 4B and C).

Endocytosis is temperature dependent [20], [33], and specifically at 4°C is fully arrested [34]. The Nb-mediated lysis gradually decreased at lowered temperature, reaching a minimum at 4°C (Fig. 4D).

We next investigated the possible influence of trypanolytic Nbs with transporter-mediated and fluid-phase endocytosis using FITC-labelled transferrin and dextran, respectively (Fig. 4E & F). Clearly, both transporter-mediated and fluid-phase uptake were reduced rapidly following the addition of lytic Nbs and this suggests that the presence of these Nbs in some manner obstructs endocytosis. Furthermore, Nb_An05 or Nb_An46 greatly reduced 2-deoxy-D-[^3^H]glucose uptake, which relies primarily on facilitated diffusion through glucose transporters, rather than endocytic activity [35], [36] (Fig. 4G). The non-lytic Nb_An33 did not have this effect. This reduced accumulation of glucose, which provides the major carbon source for glycolysis, may underlie the decline in cellular ATP at later time points when parasites are incubated with trypanolytic Nbs. The ATP levels were unaffected by non-lytic Nb_An33 (Fig. 4H). The arrest of facilitated diffusion and endocytosis occurs within a time span of ∼10 minutes, whereas the energetic crisis through ATP depletion and the loss of mitochondrial membrane potential as assayed with the cationic dye MitoPT JC-1 (Fig. 4I) are clearly a secondary effect of the presence of the trypanolytic Nbs.

**Figure 5:**
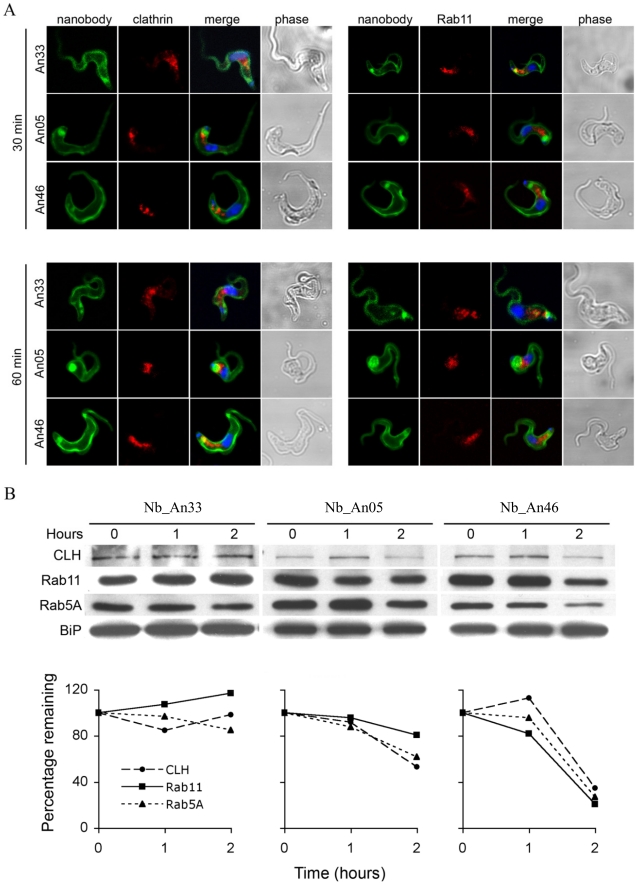
Localization and modulation in expression of endocytic and recycling markers during Nb-induced trypanolysis. A) Localization of Nbs in relation to early and recycling endosomes in *T. brucei*. Parasites (10^7^) were incubated with 10 µg ALEXA-488 labelled Nbs (Nb_An33, Nb_An05 or Nb_An46) for 30 or 60 minutes at 37°C. The ratio VSG∶Nb equals 1∶4. Fixed and permeabilized cells were counterstained with anti-clathrin antibody (red, left panels) or anti-Rab11 antibody (red, right panels). Nuclei and kinetoplast are stained with DAPI (blue). No internalised Nbs or co-localisation with anti-clathrin antibody or anti-Rab11 antibody is observed for the trypanolytic Nbs, and no major changes to the distribution of intracellular makers are observed in the presence of the Nbs. B) Upper panel, Western blots of whole-cell lysates (10^7^ cells) at different times Nb exposure using Nb_An05, Nb_An46 or Nb_An33 probed with anti-clathrin, anti-Rab5A or anti-Rab11 antibody. Lower panel, clathrin, Rab11 and Rab5A levels quantified using ImageJ from the data in the upper panel. The experiments have been performed at least twice.

Internalisation of surface VSG and fluid-phase uptake are both clathrin-mediated [23], [32], while endocytosis and recycling is regulated by Rab5A and Rab11 [8], [37]. The localization and expression of these endocytic markers was determined during Nb-induced trypanolysis. ALEXA-labelled Nb_An05 and Nb_An46 stained the entire parasite surface and accumulate in the FP, but no obvious co-localization with clathrin or Rab11 occurs after 30 or 60 minutes (Fig. 5A). Surprisingly, there is also no co-localization observed for the ALEXA-labelled control Nb_An33 with clathrin and Rab11, indicating that none of these Nbs are internalized to a detectable level. Next, protein levels of clathrin, Rab5A and Rab11 were determined after zero, one and two hours incubation with Nb_An05, Nb_An46 or control Nb_An33 by Western blotting (Fig. 5B). The protein levels of clathrin, Rab5A and Rab11 declined after incubation with Nb_An05 and Nb_An46, but remain unaffected with Nb_An33. While reduction of Rab5A, Rab11 and clathrin correlates with swelling of the FP and is in accordance with earlier data [38], it is unlikely that this represents the direct mechanism whereby endocytosis is compromised, and may rather reflect macromolecular leakage from the cells during the lysis period. The observation of a decrease of detectable Rab5 and Rab11, while BiP levels are unaffected may be due to the requirement of an extensive cell lysis; Rab5 and Rab11 are cytosolic proteins, but BiP is located within the lumen of the endoplasmic reticulum (ER).

### Trypanolytic nanobodies reconstituted into larger HCAb constructs lose trypanolytic activity

Our experiments with murine infections revealed that trypanolytic Nbs are able to control trypanosome levels (see Fig. 1E). However, camelids that produce anti-VSG HCAbs do suffer from trypanosomiasis, suggesting that the presence of anti-VSG antibody alone is insufficient for parasite control [39]. To resolve this potential contradiction we reconstituted the Nbs into a monoclonal anti-VSG HCAb molecule.

**Figure 6:**
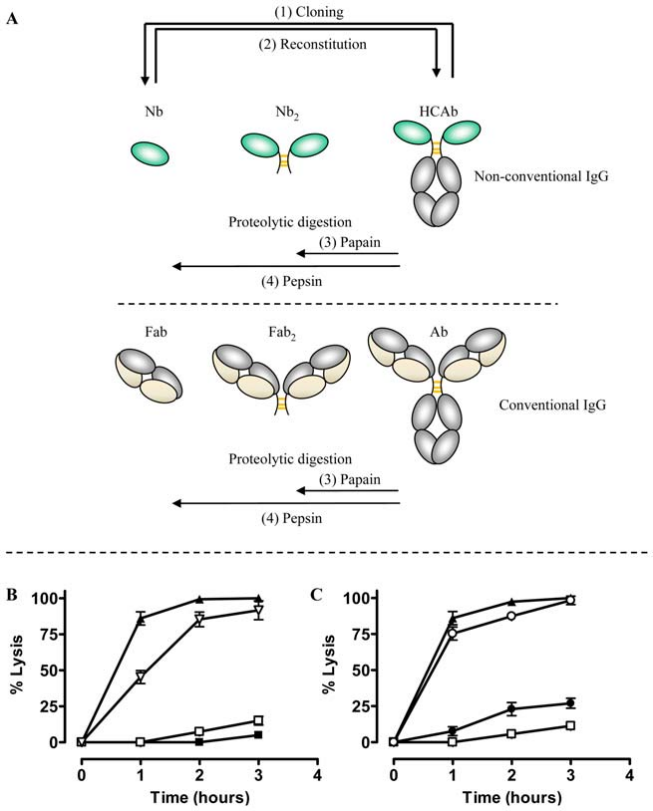
Overview of different antibody fragments and effect of reconstituted Nb_An05-Fc on *in vitro* mediated trypanolysis. (A) Schematic of the different antibody fragments. (1) Nbs are derived from the HCAbs after cloning and selection by phage-display. (2) The monoclonal Nb is reconstituted into an HCAb molecule. (3) and (4) Fab′_2_ or Nb′_2_ and Fab or Nb fragments are obtained by proteolytic digestion of conventional immunoglobulin or camelid HCAb using papain or pepsin, respectively. B) Percent lysis of monomorphic AnTat1.1 parasites over three hours at 37°C and incubated with Nb_An05 (▴), Nb_An05-Fc (□), Nb_An05-Fc and complement (∇) or without any Nb (▪). C) Percent lysis of monomorphic AnTat1.1 parasites, after incubation with recombinant Nb_An05 (▴), Nb_An05-Fc (□) or with Nb′_2_ obtained after pepsin digestion of Nb_An05-Fc (•) and Nb obtained after papain-digestion of Nb_An05-Fc (∘). The percent lysis was calculated relative to the initial parasite number. The data given are typical results from three independent experiments performed in triplicate (±SD). For each experiment 2×10^5^ parasites were incubated with 0.067 nmole Nb, Nb-Fc or Nb′_2_ constructs (VSG/antibody ratio of 1∶20).

The coding sequence for the AnTat1.1-specific and trypanolytic Nb_An05 was fused to the Fc-domain, including the hinge, of human IgG1, and this construct was transfected into NSO cells. The transfectants secrete 90 kDa Nb-Fc homodimers that lack both the CH1 domain and the light chain, and are similar to naturally occurring camelid HCAbs (see Fig. 6A upper panel (2)). Surprisingly, addition of the purified chimeric Nb_An05-Fc HCAb to AnTat1.1 trypanosomes fails to induce lysis (Fig. 6B). Nevertheless, the synthetic HCAb was perfectly functional in terms of antigen binding, bivalency and effector function as (i) Nb_An05-Fc HCAb recognised the VSG antigen by ELISA and surface plasmon resonance, (ii) addition of larger amounts of HCAb led to parasite aggregation, and (iii) addition of guinea pig complement to the trypanosomes exposed to the HCAb elicited complement-mediated parasite lysis (Fig. 6B). To confirm that the presence of the Fc-domain in the reconstituted HCAbs abolished the Nb trypanolytic activity, the monoclonal chimeric Nb_An05-Fc HCAb protein was digested with pepsin and papain to release (Nb)′_2_ and Nb, respectively (see Fig. 6A upper panel (3) and (4)). Remarkably, these proteolytic fragments regained the lytic activity towards AnTat1.1 trypanosomes, especially the monovalent Nb obtained by papain digestion (Fig. 6C).

### Trypanolytic activity of fragments derived from conventional antibodies

The data above suggested that intact immunoglobulins may possess latent functions that become apparent once the Fc and antigen-binding domains are separated. Therefore, we tested the trypanolytic activity of intact camelid serum antibodies from animals immunised with AnTat1.1 sVSG and from which the cloned Nbs were derived. Camelid serum contains two classes of IgG [27]; conventional 150 kDa antibodies consisting of light and heavy chains, and 90 kDa HCAb consisting of heavy chains only. The conventional subclass i.e. IgG1 and the HCAb subclasses, i.e. IgG2 and IgG3, of the immunised camelid were purified by differential adsorption on Protein-A and Protein-G. Antibodies in these fractions recognize purified AnTat1.1 VSG in ELISA and Western blot and also stain living *T. brucei* parasites by flow cytometry and immunofluorescence [30].

**Figure 7:**
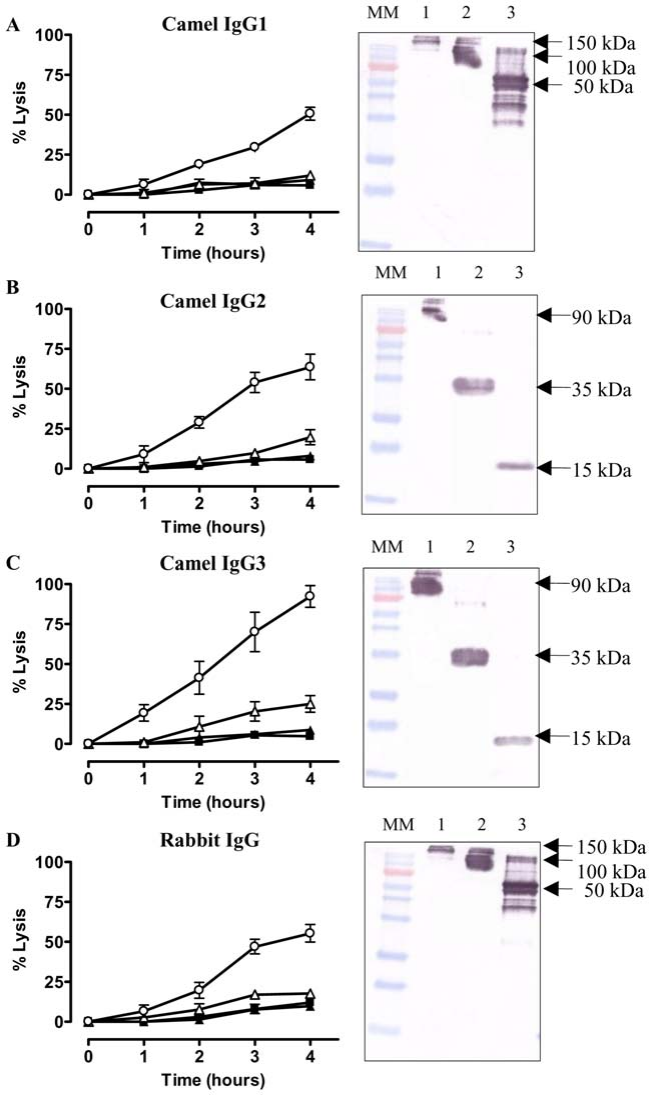
*In vitro* trypanolytic capacity of polyclonal camelid or rabbit IgGs on monomorphic AnTat1.1 parasites. Purified IgG, (Nb)′_2_, Nb, Fab′_2_ or Fab (0.067 nmole) from an immunized camelid or rabbit were added to 2×10^5^ parasites at 37°C (ratio VSG∶ antibody is 1∶20) and parasite lysis monitored over four hours. A) Percent trypanosomes lysed in the presence of VSG-specific camelid IgG1 (▴), Fab′_2_ (▵) and Fab (○). B) Trypanolysis in presence of heavy-chain only IgG2 (▴), (Nb)′_2_ (▵) or Nb (○). C) As (B) but with camelid heavy-chain antibody IgG3 and its derived fragments. D) Percent trypanosome lysis after incubation with VSG-specific rabbit IgG (▴), Fab′_2_ (▵) and Fab (○). Parasite lysis in absence of any antibody (▪). The percent lysis was calculated relative to the initial parasite number. The data are typical results from three independent experiments performed in triplicate (±SD). Each graph contains an inset of a Western blot of undigested IgG (lane 1), pepsin digested IgG (lane 2) or papain digested IgG (lane 3). (MM: 170, 130, 100, 72 (red band), 55, 40, 33, 24, 17 and 11 kDa).

Addition of the purified camelid IgG1 fraction to trypanosomes, in absence of complement, did not lyse parasites (Fig. 7A). However, proteolysis of the camelid IgG1 by pepsin and papain resulting in 100 kDa Fab′_2_ and 50 kDa Fab fragments respectively, of which only the latter demonstrated trypanolytic activity (inset Fig. 7A, right panel). Similarly, protease digestion of camelid polyclonal HCAb IgG2 and IgG3 yields bivalent 35 kDa Nb′_2_ and monovalent 15 kDa Nb antigen-binding fragments (inset Fig. 7B and C, respectively). While incubation of AnTat1.1 trypanosomes with camelid HCAbs did not result in lysis, the (Nb)′_2_ fragments elicited moderate lysis following prolonged incubation periods, while the corresponding Nb fragments provoke significant lysis (Fig. 7B and C). These results are consistent with previous observations for bivalent Nb′_2_ and monovalent Nbs derived from reconstituted Nb-Fc HCAbs (Fig. 6B).

To assess whether trypanolysis could be achieved by non-camelid antibodies, we immunized a rabbit with AnTat1.1 sVSG. The IgG fraction contained antibodies that recognized purified VSG by ELISA and Western blot (see Fig. 1 in [30]), and stained the entire surface of parasites expressing AnTat1.1 VSG (data not shown). Similarly, pepsin and papain digestions of the rabbit IgG were performed, generating Fab′_2_ and Fab, respectively (Fig. 7D, inset). The effect of the pools of polyclonal rabbit IgG, Fab′_2_ and Fab fragments in absence of complement was tested on trypanosomes *in vitro*. Only the Fab fragments lysed parasites significantly over a four hour incubation period (Fig. 7D). Collectively, these data demonstrate that generation of bivalent antigen-binding fragments suppresses the trypanolytic property of a monomeric Nb or Fab.

### Antigen binding characteristics of trypanolytic nanobodies

**Figure 8:**
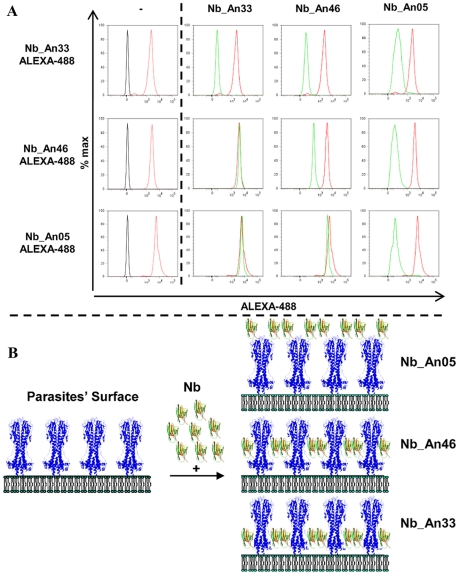
Competition binding experiments and schematic representation of the localization of Nb binding on VSG. A) Flow cytometry profiles showing binding competition among different Nbs. Left panels: profile of parasites in the absence of Nbs (black), or bound by ALEXA-labelled Nbs (red). Right panels: FACS profiles of parasites bound by ALEXA-labelled Nbs (red) or when binding of the ALEXA-labelled Nb was competed by prior incubation with unlabelled Nb as indicated at the top of the first row (green profile). B) Schematic representation of the location of the Nbs bound on GPI-anchored VSG at the parasite surface as inferred from the flow cytometry data. Nb_An33 binds close to the membrane, Nb_An05 interacts close to the membrane-distal region of VSG, and Nb_Ab46 binds at an intermediate location. Attachment of trypanolytic Nbs at the surface (Nb_An05), or slightly deeper (Nb_An46) on the VSG prevents binding of molecules to epitopes that are located more proximal to the membrane.

**Figure 9:**
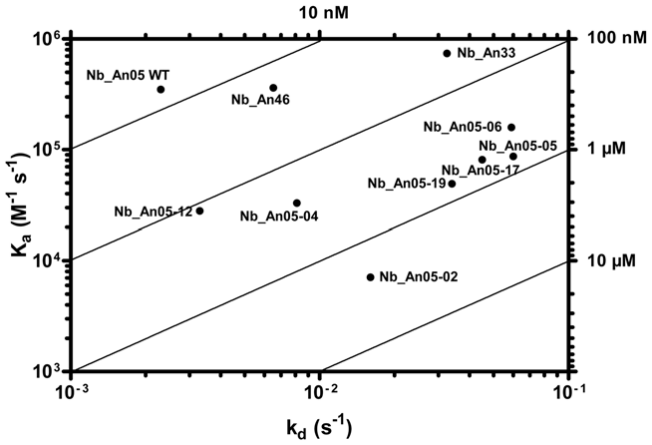
VSG recognition by Nbs. A) Rate plane with Isoaffinity Diagonals (RaPID) plot of Nb_An05 and the Nb_An05 mutants, Nb_An46 and Nb_An33 to VSG. The kinetic rate values k_on_ and k_off_ for a particular Nb to VSG determined via surface plasmon resonance are plotted on a two-dimensional graph so that pairings with identical K_D_ values are located along isoaffinity diagonals.

Besides the molecular weight and monovalency, we considered that the antigen binding properties may contribute to the trypanolytic activity. Therefore, the affinity and epitope specificity of the Nbs were analysed by surface plasmon resonance (SPR) and flow cytometry. The competitive or cumulative binding of Nbs to the AnTat1.1 antigen on intact parasites (Fig. 8A) revealed that Nb_An05 and Nb_An06 share overlapping VSG epitopes, which are distinct from the epitopes recognized by Nb_An46 and Nb_An33. The binding of the two distinct lytic Nbs, Nb_An05 and Nb_An46, to immobilised VSG occurs with comparable kinetic on-rates of 3.5 and 7.4×10^5^ M^−1^ s^−1^ respectively and more distinct off-rates of 2.3×10^−3^ and 3.25×10^−2^ s^−1^. Equilibrium dissociation constants (K_D_ = k_off_/k_on_) of 6.6, 18 and 44 nM were calculated for Nb_An05, Nb_An46 and Nb_An33 (Fig. 9 and Table 1). Interestingly, the trypanolytic Nbs exhibited smaller k_off_ values than the non-lytic Nb_An33.

**Table 1:** Antigen-binding kinetic parameters of the different Nb_An05 mutants as well as the wild type Nb_An05, Nb_An46 and Nb_An33 were determined using surface plasmon resonance (CM5/VSG-chip).

	k_on_ (1/Ms)	k_off_ (1/s)	K_D_ (nM)	% Lysis
Nb_An05-02	7.10E+03	1.60E−02	2200	0
Nb_An05-04	3.30E+04	8.10E−03	250	39
Nb_An05-05	8.70E+04	6.00E−02	690	0
Nb_An05-06	1.59E+05	5.90E−02	370	0
Nb_An05-12	2.80E+04	3.30E−03	120	13
Nb_An05-17	8.10E+04	4.50E−02	550	0
Nb_An05-19	4.93E+04	3.40E−02	690	0
Nb_An05 (WT)	3.50E+05	2.30E−03	6.6	100
Nb_An46	3.60E+05	6.50E−03	18	100
Nb_An33	7.40E+05	3.25E−02	44	0

The k_on_, k_off_ and K_D_ values and percentages trypanotolytic activity are shown for each clone. Percentage trypanolysis was determined relative to that of wild type (WT) Nb_An05 (set at 100%) and data are representative of two independent experiments. For each of these experiments 2×10^5^ parasites were incubated with 1 µg Nb (VSG∶Nb molar ratio of 1∶20).

**Figure 10:**
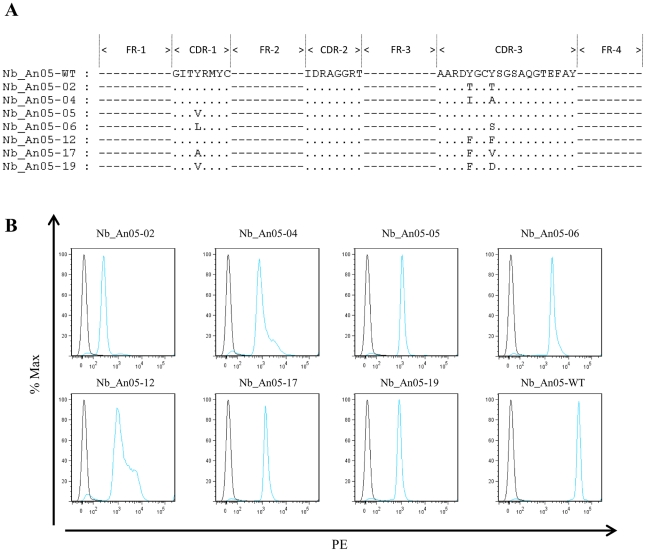
Alignment and functional characterization of Nb_An05 mutants. (A) Alignment of the Nb_An05 mutant sequences with the wild type sequence; only the mutations in the complementarity determining regions are shown. (B) Flow cytometry analysis using wild type Nb_An05 and derived variants (Nb_An05-2,4,5,6,12,17,19), detected with mouse anti-6⋅His IgG and a phycoerythrin-labeled rat anti-mouse IgG (blue line). The black line represents controls with no Nb. For each experiment 2×10^5^ parasites were incubated with 1 µg Nb (VSG∶Nb molar ratio of 1∶20).

The SPR measurements suggest that the Nb-mediated trypanolysis may only occur above a critical threshold k_off_ value (Fig. 9). To test this, the trypanolytic Nb_An05 was subjected to randomization of select tyrosine residues in its complementarity determining regions 1 and 3 (Fig. 10A). This resulted in the identification of several paratopic variants that retained trypanosome-binding, but with greatly reduced affinity ranging from 120 nM to 2 µM as compared to the 6.6 nM affinity for the wild type Nb_An05 (Fig. 10B, Table 1). Relative to the wild type Nb_An05, these variants had k_on_ rates reduced by 2 to ∼50-fold, whereas the k_off_ rates are ∼1.5 to ∼25-fold faster. Therefore the variants exhibit a wide diversity in K_D_, k_on_ and k_off_ constants. When tested against live trypanosomes, two Nb_An05 mutants, Nb_An05-04 and Nb_An05-12, retained some lytic activity against the parasite (Table 1). Remarkably, these two Nbs had the slowest k_off_ rates of all the variants. Overall, this experiment indicates that a k_off_ rate slower than 10^−2^ s^−1^ is required to detect trypanosome lysis under our current *in vitro* test conditions. The correlation between K_D_ or k_on_ and trypanolysis is less clear.

## Discussion

*Trypanosoma brucei* has evolved very efficient systems for immune evasion, which include antigenic variation and mechanisms for removal of antibody-VSG complexes from the surface by endocytosis and proteolysis of the immunoglobulin. Hereby, the VSG is efficiently recycled [8]. It is conceivable that uptake of antibody-VSG complexes and subsequent trafficking is influenced by the antibody valency and molecular weight. Exposing trypanosomes to the antigen binding domain (Fab or Nb) of an immunoglobulin alone represents a non-physiological circumstance, and the evidence presented here suggests that this presents a challenge which the parasite may be unable to circumvent.

We show that small, monovalent VSG-specific antibody fragments, Fabs or Nbs, efficiently lyse trypanosomes both *in vitro* and *in vivo*. Hereby, the monovalency of these fragments is pivotal for trypanolysis as bivalent Nb′_2_ are significantly less lytic than monomeric forms. Reconstitution of monovalent Nbs into an HCAb (increasing valency, molecular weight and incorporating an Fc-domain) abolished the trypanolytic activity *in vitro*, whereas remarkably, releasing the Nb domain via proteolysis of the recombinant HCAb restored the trypanolytic activity. This suggests that intact, bivalent monoclonal or polyclonal immunoglobulins, including rabbit and camelid classical antibodies and camelid HCAbs, are essentially harmless to trypanosomes in the absence of complement or any other bystander effector. In contrast, polyclonal Fabs or Nbs derived from the serum antibodies and deprived of classical effector Fc-domains, acquire trypanolytic activity. It should be emphasized that the trypanolytic potency of recombinant Nbs, Nbs from polyclonal HCAbs (IgG2 or IgG3), and Fabs from polyclonal IgG are not directly comparable as the exact titre of VSG-specific antigen-binding fragments within the polyclonal pool is unknown and the efficiency of lysis is clearly concentration dependent (Fig. 1D).

Immunoglobulins evolved with the antigen binding site (Fab) at one pole and with Fc effector functions that trigger complement-mediated killing and receptor-mediated phagocytosis at the other. Normally these effector functions are exerted only following antigen binding and are mediated by the Fc-domain. Nevertheless, intrinsic activities within antigen-binding domains may be present. For example Nbs with competitive enzyme inhibiting capacity [40], [41] or Fabs with catalytic activity, ‘Abzymes’, have been described [42], [43]. In addition, Fabs can exhibit an intrinsic ability to convert molecular oxygen into hydrogen peroxide, which may contribute to destruction of the bound antigen [44], [45]. This latter activity cannot be at the origin of the trypanolytic activity described here, as hydrogen peroxide formation occurs in the hydrophobic cavity between the VH and VL domains and reduction of singlet oxygen is catalysed by VH residues Trp-36 and Trp-47 [46]. These conditions are absent in Nbs, which lack a VL-domain, while Trp47 is also substituted. Moreover, we were unable to detect hydrogen peroxide formation during trypanolysis (data not shown).

Despite the highly similar phenotypes following trypanolytic Nb exposure and RNAi of specific endocytic factors [32], [38], [47], two very important differences suggest distinct mechanism. Firstly, the Nbs elicit FP enlargement much more rapidly than RNAi, and too quickly for this to be possible via turnover of critical proteins. Secondly, the Nb is an exogenous agent. Other small exogenously delivered molecules, including aptamers [48], cathelicidins [49], neuropeptides [50] and a modified bovine host defence peptide (BMAP-18) [51] can also elicit trypanolysis in the absence of any bystander or toxin, but their modes of action are clearly distinct from the Nbs. For example cathelicidins disrupt surface membrane integrity, which is preceded by immobilisation and rapid swelling of the parasite. Although immobilisation and swelling of the parasites also occurs with Nbs, the outer membrane integrity is not significantly affected as evidenced flow-cytometrically by the lack of leakage of FITC-labelled Dextran (4 kDa) when parasites are incubated with trypanolytic Nbs. Therefore, with Nbs it seems that reduction in ATP-levels, with effects on motility, endocytosis and morphology, rather than direct surface membrane disruption, is a crucial step in parasite killing. The rapid and very severe block to endocytosis is remarkable and multiple lines of evidence demonstrate this; including accumulation of lytic Nbs in the FP and impairment to removal of the VSG-bound Nb from the parasite surface compared with non-lytic Nbs and IgG. The absence of intracellular staining or co-localisation with clathrin or Rab11 with any trypanolytic Nb strongly suggests that Nbs are not internalized to any significant degree, but temperature dependence suggests that this is an active process. It is possible that the protection accorded by lower temperature is due to inhibition of membrane transport, so preventing the FP enlargement. Drastic swelling of the FP is indicative of a block to bulk membrane endocytosis, in the presence of ongoing exocytosis, and has been reported previously in energy-depleted cells [52]. Furthermore, MitoPT™ JC-1 staining detected depolarization of the mitochondrial membrane at later times after trypanolytic Nbs exposure, but given an absence of intracellular Nbs, no direct effect on the mitochondrial membrane can be assumed.

It is difficult to pinpoint the critical parameter responsible for the intrinsic destructive capacity of the monovalent, antigen-binding fragments and where a bivalent character or the presence of the Fc-domain is counterproductive for trypanolysis. Several factors are likely important, although they probably act synergistically in attaining lytic activity. Firstly, to be trypanolytic Nbs must bind VSG with high affinity. Mutagenesis-derived trypanolytic Nb_An05 variants that recognize the same epitope with modified binding kinetics indicate that toxicity requires slow release kinetics (low k_off_), suggesting that prolonged interaction with VSG is beneficial to lysis. However, as monovalency dominates the binding parameters it is not possible to increase trypanolytic potency with bivalent constructs. Secondly, the Nb-VSG complex, unlike the IgG-VSG complex where the IgG potentially cross-links two VSG dimers, is not internalized and therefore remains at the surface. Engstler et al [12] found that smaller antibody fragments have reduced clearance from the parasite surface compared to intact antibodies and our results indeed confirm that there is greatly reduced VSG-Nb elimination from the surface; however in the case here we also find a failure to be internalized into the parasite cell. Third, the precise VSG epitope targeted by the Nb is likely important. Interestingly, some epitopes including the conserved N-glycan present on various VSG serotypes and recognized by Nb_An33 [30], [31] failed to induce lysis, whereas Nb_An46 and Nb_An05, targeting different epitopes, induced potent lysis. Remarkably, the competition binding experiments suggest that the most potent trypanolytic Nb has a binding site furthest from the membrane and may even occlude access of molecules to underlying epitopes (see Fig. 8B). Fourth, the observation that parasites in presence of trypanolytic Nbs have reduced ATP levels suggests a correlation between energy-depletion and reduced endocytosis. Fifth, the observation that parasites in presence of trypanolytic Nbs exhibit a loss in mitochondrial membrane potential (Δψ_m_) likely contributes to the observed reduced ATP levels. Sixth, the impaired flagellar motility observed very rapidly and only in presence of trypanolytic Nbs might be a crucial initiation step in the trypanolysis process [53].

Of the different mechanisms by which trypanolytic Nbs could cause lysis, the model we favour is that high affinity binding (mediated by a low k_off_) of trypanolytic Nbs to VSG impairs recycling of the surface, and within minutes this translates into impaired cellular motility. This rapidly blocks formation and/or budding of clathrin-coated pits, i.e. endocytosis. The swelling of the FP is likely to lead rapidly to cell lysis, which was also observed using clathrin RNAi. It is however unlikely that this process itself leads to decreased ATP, as ATP levels decrease more slowly than the onset of cellular defects. This slow loss of low molecular weight ATP (509 Da) also effectively eliminates the possibility of rapid generation of pores or disruptions in the plasma membrane, although we cannot rule out possible smaller disruptions to the lipid bilayer that could result in ionic imbalance for example. Further, we also observed very rapid loss of glucose accumulation, but as glucose is mainly accumulated through GLUT channels in the bulk plasma membrane, it is unlikely that this is directly related to decreased endocytosis. One possible explanation for the decreased glucose transport across the plasma membrane is that lower endocytic activity and motility reduce the draw on ATP and hence glucose utilization, decreasing the concentration gradient for glucose transport into the cell, and hence lowering glucose uptake. It may also be that this reduced consumption masks an otherwise more prominent change to intracellular ATP levels. Therefore trypanolytic Nbs in some manner are able to compromise cellular energetics, but the connection between binding VSG, compromised endocytosis and lower cellular energy remains unclear. In contrast, Nb_An33 binds to a sugar epitope and therefore does not cause the above described phenotype. Furthermore, given that Nb_An33 has a higher koff-value means that it dissociates faster from the coat so that it can not exert a trypanolytic effect.

In conclusion, the present work demonstrates firstly that high affinity antigen-binding antibody fragments can exert a direct biological “cytotoxic” function in the absence of the effector Fc-domain, and which is latent in intact immunoglobulins. Secondly, targeting the trypanosome surface with such small high affinity antigen-binding fragments is sufficient to efficiently kill the parasite. In addition, Nbs targeting various epitopes on the surface coat of trypanosomes offers possibilities for novel treatments for trypanosomiasis by developing small trypanotoxic compounds that compromise cell viability. Indeed, since the lytic Nb_An05 and Nb_An46 recognize distinct AnTat 1.1-specific peptidic epitopes, it seems that multiple sites on VSG could serve to target therapeutics. Moreover, the observation that Nb_An05 and Nb_An06 have overlapping epitopes that are not necessarily identical (these Nbs have different CDR sequences) suggests that the therapeutic epitope could be reduced in size to a small footprint of only a few hundred Å^2^ that might be conserved among VSGs of various serotypes. Although the specificity for a particular VSG, as is the case for the trypanolytic Nbs here imposes a limitation on therapeutic value, our data indicate that a cross-reactive therapeutic Nb recognizing many or all VSGs would require binding at a conserved VSG epitope with very high affinity. Under this assumption it might become feasible to design or select small organic compounds that would bind with high affinity to a VSG epitope, leading to trypanosome clearance, and as such be used as a novel therapeutical approach.

## Materials and Methods

### Ethics statement

The experiments, maintenance and care of mice complied with the guidelines of the European Convention for the Protection of Vertebrate Animals used for Experimental and other Scientific Purposes (CETS n° 123). The experiments for this study were approved by the Ethical Committee for Animal Experiments of the Vrije Universiteit Brussel, VUB, Brussels, Belgium (Permit Number: 08-220-8).

### Parasites, VSG and antibody preparations

Purification of *Trypanosoma b. brucei* (AnTat1.1, MiTat1.1, MiTat1.2, MiTat1.5 and MiTat1.6) bloodstream parasites, their soluble VSGs and the immunization of a camelid with AnTat1.1 sVSG was as described [30]. Camelid serum IgG fractionation, cloning, selection and purification of Nbs was according to published methods [30], [54], and reconstitution of HCAbs by fusing the Nb_An05 or Nb_An33 to the human Fc of IgG1 was as explained in [55].

### Digestion of immunoglobulins with pepsin or papain

Purified IgG was digested with 1% Hg-papain (Sigma) or porcine pepsin (EC 3.4.23.1, Sigma) following the manufacturer's instructions. The digest was passed over Protein-G Sepharose and gel-permeation Superdex-200 (10/30) (GE Healthcare) in PBS (pH 7.4) to purify Fab, Fab′_2_, (Nb)′_2_ or Nb. The protein concentration was assessed spectrophotometrically.

### Western blot analysis

From each antibody, or digested material, 130 pmole was loaded onto a 12% SDS-polyacrylamide gel (under non-reducing conditions), and transferred to nitrocellulose. After blocking with 1% (w/v) bovine serum albumin, the membrane was incubated sequentially with a rabbit polyclonal anti-VHH IgG and a goat anti-rabbit-IgG antibody conjugated to horseradish peroxidase (Sigma). In between the successive two hour incubations was a PBS-0.1% Tween 20 wash. Thirty minutes after adding chromogenic substrate (methanol/4-chloro-1-nafthol in PBS/H_2_O_2_) the reaction was stopped by rinsing the membrane with water.

The determination of the protein expression levels of Clathrin, Rab5A, Rab11 and BiP during the trypanolysis assay was performed as described elsewhere [56]. Chemiluminescense detection was by exposure to X-ray film (Kodak BioMax MR), and ImageJ software used for quantification.

### Affinity measurement of Nbs

For the affinity determination with Biacore 3000, different concentrations, ranging from 500 nM to 7.5 nM, of Nb_An05, Nb_An06, Nb_An46 or Nb_An33 were added to a CM5 chip to which 500 RU of AnTat1.1 VSG had been coupled [57]. Sensograms were fitted for a 1∶1 binding model using the BIA-evaluation software version 4.1 (GE Healthcare), resulting in k_on_, k_off_ and K_D_ values as output.

The affinities of the Nb_An05 mutants were measured by surface plasmon resonance on a Biacore T100 system. Between 1000 and 1500 RU of soluble AnTat1.1 VSG was coupled onto a CM5 chip (GE Healthcare) via amine groups according to the manufacturer's descriptions using EDC and NHS as cross-linking agents and ethanolamine to block free esters. For the affinity determination, Nb concentrations ranging from 500 to 7.5 nM were added to the antigen-coated chip at a flow-rate of 30 µl/min in HBS buffer [10 mM Hepes (pH 7.5), 150 mM NaCl, 3.5 mM EDTA and 0.005% (v/v) Tween-20)]. Bound Nbs were eluted with 10 mM glycine-HCl (pH 2.5). Sensograms were fitted for a 1∶1 binding model using the Biacore T100 Evaluation Software 2.0.2 (GE Healthcare), calculating k_on_, k_off_ and K_D_ values.

### Flow cytometry analysis

The different Nb clones were evaluated on live, bloodstream form AnTat1.1 trypanosomes through flow cytometry following a direct or three-step labeling procedure. The direct labeling required conjugation of Nbs with ALEXA Fluor 488 according to the manufacturer's instructions (Molecular Probes). Hereby, parasites (2×10^5^ in 100 µl PBS/10% FCS) were cooled in an ice-bath (30 minutes) before adding Nbs. After 10 minutes incubation with ALEXA-labelled Nbs (1 µg), cells were washed with ice-cold PBS/10% FCS and analyzed. The three-step labeling procedure relied on the detection of the surface-bound Nbs with a mouse anti-6⋅His IgG and a phycoerythrin-labeled rat anti-mouse IgG. Flow cytometry analyses were performed on a FACS Canto II and histograms were prepared using the FlowJo software (Becton Dickinson, San Jose, CA).

To evaluate the antibody-clearance rate by trypanosomes, a pulse-chase experiment was performed. This consisted of incubation of 2×10^5^ parasites with 1 µg ALEXA-labelled Nbs (Nb_An05, Nb_An46, Nb_An33 or irrelevant Nb) or 10 µg rabbit polyclonal IgGs against VSG for 10 minutes on ice in HMI-9 medium/5% FCS. Next, the free antibodies were washed away by washing the parasites 2 times with ice cold HMI-9 medium. The parasites were resuspended at 5×10^6^/ml in HMI-9/5% FCS in separate tubes and brought at 37°C. At different time-points (0-0.5-1-1.5-2.5-5-7.5-10-30-60-120 minutes), aliquots were washed with 2 ml HMI-9 to remove free antibodies. The parasites were resuspended in 100 µl HMI-9 followed by addition of 100 µl 4% paraformaldehyde/PBS to stop the metabolic activity. Following a 30 minutes fixation step, the cells were washed with ice-cold PBS/10% FCS and analyzed as described above. The mean-fluorescence intensity of parasites incubated with the antibody at time 0 was taken as the 100 percent signal.

### Immuno-fluorescence microscopy

Nbs were labelled with ALEXA-488 (Molecular Probes) according to the manufacturer. Aliquots of 10^6^ parasites were incubated with 10% normal rabbit serum in PBS for 30 min in an ice-bath before adding different ALEXA-labelled Nbs (1 µg), ALEXA-labelled rabbit polyclonal anti-VSG IgG (5 µg), camelid polyclonal anti-VSG IgG (5 µg) or Nb_An-Fc chimer (6 µg). After 30 minutes the parasites were pelleted, washed with 10% normal rabbit serum in PBS, and analysed by fluorescence microscopy (Nikon ECLIPSE E600 with phase contrast, 500×–1250× magnification).

To assess the role of the membrane fluidity in uptake of Nbs, parasites were pre-incubated for 1 hour at 4°C or 37°C before adding ALEXA-labelled Nb_An05 or control Nb. After 30 minutes, parasites were washed 3 times with PBS/5% FCS and analysed by immuno-fluorescence microscopy. Individual samples were taken every 30 minutes and visualised by fluorescence microscopy to study the kinetics of Nb clearance by parasites. The co-localization experiments were performed as described [56], [58]. Images were obtained using a Nikon ECLIPSE E600 epifluorescence microscope fitted with optically matched filter blocks and a Hamamatsu charge-coupled-device camera or a Leica confocal laser-scanning microscope. Images were false-coloured and assembled using Adobe Photoshop.

### Trypanolysis assays

*In vitro:* short term *ex vivo* trypanolysis was performed using 200 µl DEAE52-purified parasites (stock: 10^6^ parasites/ml HMI-9 medium/5% FCS) which were incubated at 37°C at 5% CO_2_ in a humidified atmosphere with different antibodies (rabbit polyclonal anti-VSG, Fab, Fab′_2_, fractionated polyclonal camelid IgG, Nbs, (Nb)′_2_ or Nb_An-Fc) at a maximum concentration of 0.067 nmole. The surviving parasites were counted at regular intervals over a time period up to 5 hours using a Bürker hematocytometer. For the inhibition of the Nb-mediated trypanolytic activity, the Nbs were pre-incubated for 30 minutes with a 3-times molar excess of purified AnTat1.1 soluble VSG, prior to addition to the parasites. The percentage lysis was calculated relative to the condition with a non-trypanosome specific Nb or without Nb. For the site-directed mutagenized Nb_An05 trypanolysis experiments, 2×10^5^ parasites in 200 µl HMI-9 medium supplemented with 10% decomplemented fetal bovine serum (FBS) were incubated with 1 µg Nb, followed by incubation at 37°C in a conditioned atmosphere with 5% CO_2_ for 5 hours. Lysis was quantified by parasite counting using a Bürker hematocytometer and the percentage lysis of the different paratope variants was calculated relative to that of wild type Nb_An05 (i.e. 100 percent).

*In vivo:* Eight-weeks old F1-mice were injected intra-peritoneally (i.p.) with 5000 virulent monomorphic AnTat1.1A parasites per mouse. Starting from day 1 till day 4 post infection, 100 µg Nb was i.p. injected. The parasitemia in 2.5 µl blood (obtained from the tail vein of infected mice) diluted in 500 µl PBS was monitored microscopically, and the survival of the mice was recorded.

### Mechanism of VHH-mediated trypanolysis

Parasites (2×10^5^ in 200 µl HMI-9 medium/5% FCS) were incubated for 1 hour at 37°C, 25°C, 15°C and 4°C to reduce or stop the membrane fluidity, before adding Nbs (1 µg) and to monitor their survival over a 4–5 hour period.

The interference from Nbs on the specific or non-specific uptake of nutrients by trypanosomes was assessed by adding FITC-labelled transferrin or dextran (Sigma), respectively. After a total of 10 minutes incubation, parasites were pelleted, washed 3 times with HMI-9 medium/5% FCS, suspended in PBS and FITC-labelled nutrient uptake monitored by fluorescence readings (Cytofluor II, PerSeptive Biosystems).

The 2-deoxy-D-[1-^3^H]glucose (1 mM, 1 µCi, Perkin Elmer) uptake by 2×10^6^ parasites in presence of 2 µg Nbs was as described in [59]. Cells were lysed and the glucose concentration determined by triplicate measurements using a liquid scintillation beta-counter (Perkin-Elmer Rackbeta, Boston USA). The total protein concentration was determined as described in [60]. The data are expressed as percentage of glucose uptake relative to the glucose-level of parasites incubated for the same time period without Nbs.

To determine the effect of endocytosis disturbance by Nbs (10 µg) on the internal ATP concentration, 10^7^ parasites (at 37°C) were lysed after different time intervals by three freeze-thawing cycles. The ATP concentration of triplicate samples was quantified by the ATP-assay (Molecular Probes).

The Mitochondrial Permeability Potential (Δψ_m_) was determined using the cationic dye MitoPT™ JC-1 (Immunohistochemistry Technologies, Bloomington, MN), which exhibits potential-dependent accumulation in mitochondria. At low membrane potentials, JC-1 continues to exist as a monomer and produces a green fluorescence (emission at 527 nm). At high membrane potentials or concentrations, JC-1 forms J aggregates (emission at 590 nm) and produces a red fluorescence. The staining procedure was as recommended by the suppliers and the trypanolysis assay was performed as described above. Briefly, after different time points of the trypanolysis assay a 1∶1 ratio of the MitoPT JC-1 staining solution was added and the cells incubated for an additional 15 minutes in a CO_2_ incubator at 37°C. Next, the cells were pelleted and washed twice with 2 ml assay buffer warmed at 37°C. Finally, the fluorescent signals were measured by flow cytometry. As positive control, the cells were incubated with a final concentration of 50 µM Carbonylcyanide m-chlorophenylhydrazone (CCCP) for 60 minutes in a CO_2_ incubator at 37°C.

### Electron microscopy morphology studies

For electron microscopy cells were prepared as described elsewhere [38]. Observations were made on a Tecnai 10 electron microscope and images were captured with a MegaView II camera and processed with AnalySIS and Adobe Photoshop software.

The co-localization experiments were performed as described in [56]. All manipulations were conducted using HMI-9/5% FCS. Parasites (10^7^/ml) were incubated with ALEXA-labelled Nbs (10 µg/ml) at 37°C for 0–30 or 60 minutes followed by two washes with PBS and fixed with 4% paraformaldehyde (PFA) in ice-cold PBS. Immunofluorescence was performed as described in [58] with a few modifications. Using an ImmEdge pen (Vector Laboratories, Burlingame, CA), compartments were drawn on a poly-lysine slide (Polysine; VWR International, Leuven, Belgium) and 200 µl of 4% PFA-fixed cells was placed in each compartment. The slides were incubated in a moist chamber, and the cells were allowed to settle on the slide followed by a permeabilisation with 0.1% Triton X-100. Staining was performed as described previously [58]. The trypanosomal Golgi complex was stained using dapi (Vectashield). Cells were observed either on a Nikon Microphot-FX epifluorescent microscope attached to a Photometrics CH350-CCD camera or with a Laser Scanning Microscope 510 (Zeiss). Images were false-coloured and assembled using Adobe PhotoShop.

To follow the kinetics of ALEXA-labeled Nb uptake, parasites were incubated (37°C) in presence of ALEXA-labelled Nbs over a 2 hours time period. Next, parasites were washed twice with PBS/5% FCS, fixed with PFA in ice-cold PBS and processed as described above.

### Generation of nanobody 05 (Nb_An05) variants

Variants of Nb_An05 were generated by site-directed mutagenesis using degenerate primers (5′CCGGCCATGGCCGATGTGCAGCTGGTGGAGTCTGGGGGAGGCTCGGTA CTAACTGGAGGGTCTCTGAGACTCTCCTGTGCAGCCCCTGGAATCACCNHTCGTATGTACTGCATGGCC-3′ and 5′-GGAGACGGTGACCTGGGTCCCCCGGCCCCAGTAA GCAAACTCAGTTCCCTGAGCGGAGCCTGAADNGCAGCCADNGTCTCTTGCCG-3′) which allow replacement of tyrosine residues in complementarity determining regions (CDR) 1 and CDR3 that are anticipated to contribute in the interaction with the VSG-antigen. The PCR amplicons were subsequently cloned into pMES using PstI and BstEII restriction sites and individual mutant clones were screened for their functionality in a VSG-specific ELISA, using a peroxidase conjugated anti-6×His detection IgG (Serotec).

### GenBank accession numbers

Nb_An05-02 (HQ680967), Nb_An05-04 (HQ680968), Nb_An05-05 (HQ680969), Nb_An05-06 (HQ680970), Nb_An05-12 (HQ680971), Nb_An05-17 (HQ680972), Nb_An05-19 (HQ680973).
